# Canine hip dysplasia screening: Comparison of early evaluation to final grading in 231 dogs with Fédération Cynologique Internationale A and B

**DOI:** 10.1371/journal.pone.0233257

**Published:** 2020-05-18

**Authors:** Roxana Merca, Barbara Bockstahler, Aldo Vezzoni, Alexander Tichy, Simona Boano, Britta Vidoni

**Affiliations:** 1 Department for Companion Animals and Horses, University Clinic for Small Animals, Small Animal Surgery, University of Veterinary Medicine, Vienna, Austria; 2 Clinica Veterinaria Vezzoni, Cremona, Italy; University of Bologna, ITALY

## Abstract

**Objectives:**

This study aimed to verify if a significant difference exists between parameters in the early evaluation of normal and near-normal hip joints, to evaluate the influence of age and breed on the parameters, and to clarify the usefulness of a total score for differentiating between Fédération Cynologique Internationale (FCI) grade A and B hips.

**Methods:**

A total of 231 dogs were classified according to whether they had FCI A or B hips at adulthood, with measurements obtained at 14–28 weeks of age. The total score was calculated by the summation of the following quantitative parameters: angle of subluxation (AS), angle of reduction (AR), laxity index (LI), and dorsal acetabular rim slope (DARS). Logistic regression analysis was performed to establish the probability of the study population to develop an FCI B hip based on the total score. This was repeated for the highest score in combination with the worst-rated hip and once more for breeds.

**Results:**

No correlation between age and the parameters was found in the cohort, or for FCI A and B. The values of all the parameters were significantly lower in the FCI A group than in the FCI B group (AR: 4.42° ± 6.0° vs 7.62° ± 7.2°; AS: 0.45° ± 1.9° vs 1.55° ± 3.8°; LI: 0.32 ± 0.1 vs 0.36 ± 0.1; DARS: 3.30° ± 1.8° vs 3.77° ± 1.9°; TS: 11.47 ± 8.3 vs 16.65 ± 10.9). Labrador Retrievers and Golden Retrievers showed significant differences between parameters for both FCI grades. The range, where FCI A and B hips can be predicted on the basis of the total score, was different when assessed for the entire cohort, Labrador Retrievers, and Golden Retrievers.

**Clinical significance:**

Our results show that even in normal and near-normal hips, the parameters significantly differed in the early evaluation. Moreover, cutoff values should be set for different breeds in the prediction of the FCI grade during early evaluation for a better breeding selection regarding canine hip dysplasia, one of the most common orthopedic diseases among large and giant breed dogs.

## Introduction

Canine hip dysplasia (CHD) is one of the most common non-traumatic orthopedic diseases in large and giant breed dogs [1,2]. The pathogenic process involves hip laxity, incongruence of the coxofemoral joint, synovitis, abnormal progression of endochondral ossification and development of osteoarthritis (OA) [3,4], manifesting as restricted joint mobility, pain, and lameness. Etiological roles for genetic and environmental factors have been described in the CHD literature. Some influencing factors such as physical activity, dietary factors or body weight can be readily modulated by the owner [2,5–9], but not other factors such as an anomalous inclination of the dorsal acetabulum or hormonal influences [9–13]. Physical and therapeutic interventions increase the cost of lifetime care (for both pet and working dogs) and, if unsuccessful, raise the option of euthanasia [13,14].

Although numerous CHD screening programs that allow phenotypic selection are common in breeding clubs, only moderate success has been achieved for the improvement of the incidence of CHD [15–22]. The distinction between Fédération Cynologique Internationale (FCI) grade A and B hips is given by the Kennel clubs through their breeding regulations. In several breeds, dogs that have been evaluated as having FCI B hips are only allowed to pair with dogs that have been evaluated as having FCI A hips [23]. To our knowledge, no studies have specifically focused on the development of arthrosis in FCI A hips; however, Belgian shepherd dogs with borderline hip dysplasia have been shown to have altered joint kinematics [24]. Early evaluation (EE) of the hip joint remains a key clinical predictor of the outcome at the final evaluation performed upon skeletal maturity [9,14–19,25]. Thus, EE enables decision making for early surgical preventive intervention in growing dogs [1,26–29] or for timely exclusion from breeding or training programs. However, no evidence-based consensus has been reached about the precise method of EE, or the parameter profile or hierarchy for such decision making [30]. Furthermore, the thresholds and breed variations of the parameters for the prediction of the final FCI score remain undefined [19,31].

The objective of this retrospective study was to verify whether EE can reveal differences in the parameters, including laxity index (LI), dorsal acetabular rim slope (DARS), femoral head center (FHC), angle of subluxation (AS), and angle of reduction (AR), between hips later classified as FCI A or B. The study also evaluated the influences of age and breed on the different parameters, and verified the probability of differentiating between later FCI A and B classifications to facilitate early breeding selection and training purposes.

## Materials and methods

The medical records in Clinica Veterinaria Vezzoni were searched for dogs with data obtained at 14–28 weeks of age at EE, without surgical preventive intervention for CHD, with FCI A or B hip at 12 months or older, and both hips evaluated separately. A total of 231 dogs (462 evaluated hips) were included in the study. The study population included mixed- and pure-breed dogs. For the evaluation of breed influence, the cohort was subdivided into breeds. All the groups with <10 dogs (with low statistical relevance) were excluded. The breeds evaluated were as follows: Border Collie, Bernese Mountain Dog, Golden Retriever and Labrador Retriever.

Clinical and radiographic examinations were performed either by a surgery or imaging resident or by a board-certified surgeon. For all the dogs, EE was performed under deep sedation and included a quantitative evaluation of the Ortolani sign based on the measurements of AS and AR. The measurements were performed in dorsal recumbency using the Slocum Electronic Goniometer (Slocum Enterprises Inc., Eugene, OR, USA).

Radiographs were taken for the extended ventrodorsal, dorsal acetabular rim and distraction views [32]. The resulting parameters included LI [33], DARS [34] and FHC [35]. FHC was evaluated in the ventrodorsal view and classified as medial, superimposed, or lateral to the dorsal acetabular rim. DARS was obtained using the technique described by Slocum [34]. The distraction view was obtained as originally described by Badertscher [32], using the Vezzoni modified Bardertscher distension device (VMBDD) [33,36]. LI was measured in accordance with the method used by Smith and colleagues [8,33,37–39]. After reaching skeletal maturity, all the dogs underwent CHD assessment in accordance with FCI standards [40],by a veterinarian with FCI board certification. CHD assessments according to FCI standards have been performed in the ventrodorsal radiographic view.

Data were evaluated using the SPSS version 24 statistical software (IBM Corp., Armonk, NY, USA). A total score (TS) was obtained, similar to the global index published before [19], represented by the sum of all quantitative parameters evaluated [AS+AR+(LI × 10)+DARS], where the LI is multiplied by 10 to account for its value being always below 1. The FHC parameter was qualitative and could therefore not be included in the TS. Dogs that tested negative for the Ortolani sign were included in the statistical evaluation, with a value of 0 for both AR and AS.

The study population was divided into two groups (A and B) according to FCI grade. Logistic regression analysis was performed to establish the probability of development of grade B hips based on the TS. The left and right hips were evaluated separately. This was repeated for the highest score of each dog in combination with the worst-rated hip of each dog. Thus, in all the logistic regression analyses, the statistical unit was the dog. In addition, we performed these analyses for breed groups with >10 dogs. Odds ratios, including 95% confidence intervals, were calculated for the logistic regression using the highest TS of each dog.

The Student *t* test was used for the comparisons of AS, AR, LI, and DARS between the FCI A and B groups. A Pearson correlation analysis was performed to assess the correlation between age and the parameters. The chi-square test was used to evaluate the differences in FHC between groups A and B. The effects of group and breed on the different parameters were analyzed using a general linear model with the Tukey alpha correction procedure as a post hoc test for breed. The assumption of normal distribution was assessed using the Kolmogorov-Smirnov test. A *p* value of <0.05 was considered statistically significant.

## Results

### Demographics

The female-to-male ratio was 1:1 (50.6% females and 49.4% males). The mean age was 20.7 ± 3.4 weeks at EE and 64.3 ± 20.6 weeks at final FCI examination, with 303 FCI A and 159 FCI B hip joints. The most frequently represented breeds were Labrador Retriever (n = 93, 40.3%), Border Collie (n = 36, 15.6%), Golden Retriever (n = 36, 15.6%), and Bernese Mountain Dog (n = 31, 13.4%). A total of 216 hips tested positive for the Ortolani sign, with AR ranging from 5° to 30°. The values (mean ± SD) of the parameters in the entire study population were as follows: AR, 5.52° ± 6.6°; AS, 0.83° ± 2.7°; LI, 0.33 ± 0.1; DARS, 3.46° ± 1.8°; and TS, 13.25 ± 9.6. The FHC was located medial to the acetabular rim in 408 hips (88.3%), superimposed in 52 (11.2%) and lateral to the acetabular rim in 2 (0.4%).

### Correlation analysis

While no correlation was found between the age of the subjects and the evaluated parameters, a significant correlation was found among the evaluated parameters (Table 1).

**Table 1:** Correlation (r) of the parameters with age and among the parameters in the entire population.

r	AR	AS	LI	DARS
**age**	−0.05	0.04	0.02	−0.08
**AR**		0.46a	0.49a	0.32a
**AS**			0.33a	0.21a
**LI**				0.36a

**All the correlations marked with *a* revealed a *p* value of** <0.001.

The AR and TS showed the strongest correlation, whereas DARS and TS showed the weakest correlation.

### Correlations of the FCI A and B groups in the entire population

**Fig 1:**
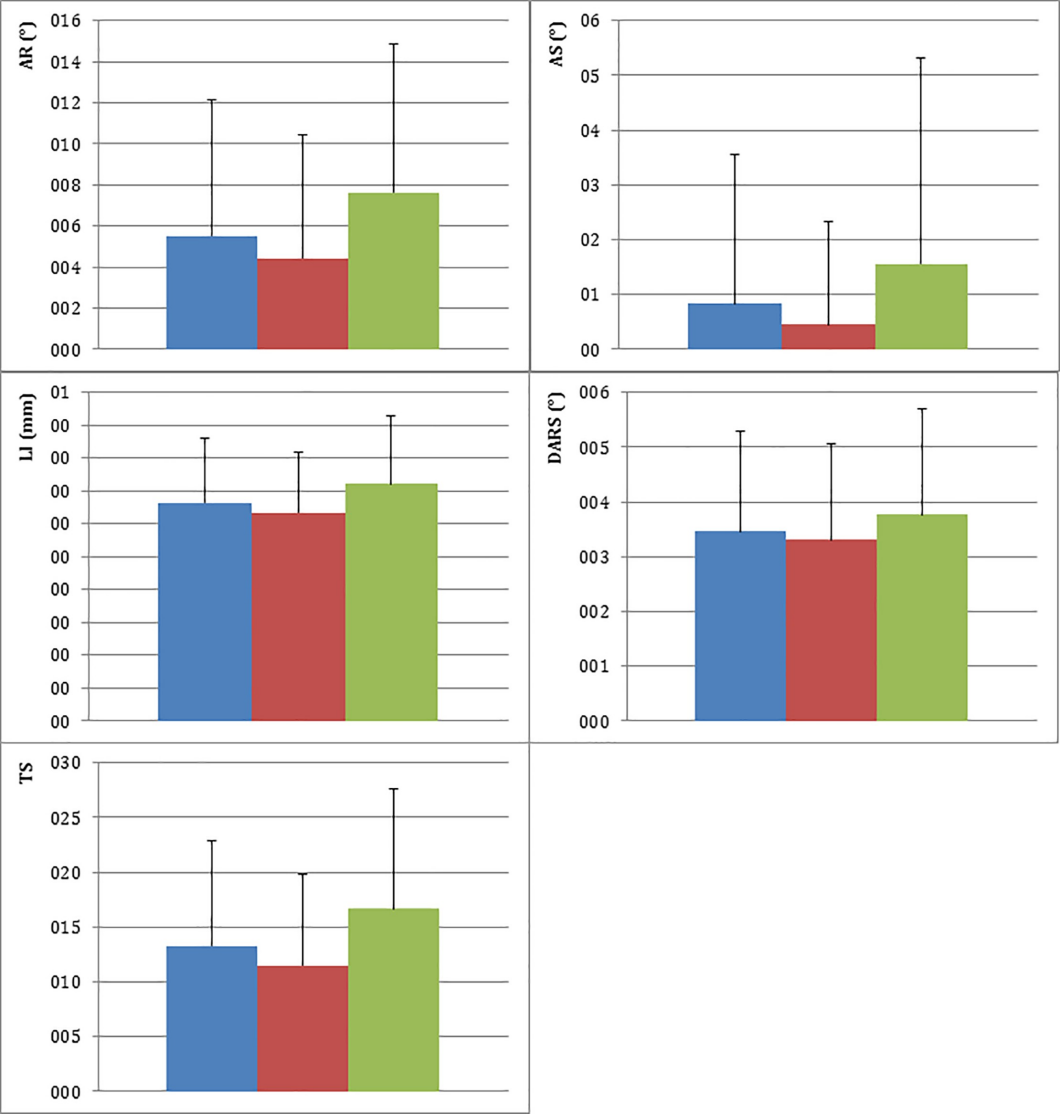
Values of the parameters at the time of early evaluation. 1: AR (°), 2: AS (°), 3: LI, 4: DARS (°), and 5: TS. The blue bars represent the entire population: the red bars, the FCI A group: and the green bars, the FCI B group. * Significant difference in the evaluated parameters between the FCI A and B groups.

The values (mean ± SD) of the parameters evaluated at EE for the hips with adult grades of FCI A and B were as follows: AR, 4.42° ± 6.0° vs 7.62° ± 7.2°; AS, 0.45° ± 1.9° vs 1.55° ± 3.8°; LI, 0.32 ± 0.1 vs 0.36 ± 0.1; DARS, 3.30° ± 1.8° vs 3.77° ± 1.9°; and TS, 11.47 ± 8.3 vs 16.65 ± 10.9. In the FCI group, the FHC at EE was located medial to the acetabular rim in 278 hips (91.7%), superimposed in 24 (7.9%), and lateral to the acetabular rim in 1 (0.3%). In the FCI group, the FHC at EE was located medial to the acetabular rim in 130 hips (81.8%), superimposed in 28 (17.6%), and lateral to the acetabular rim in 1 (0.6%). The values of all the parameters tested at EE were significantly lower in the FCI A than in the FCI B group (*p* < 0.001 for AR, AS, LI, and TS, and *p* = 0.01 for DARS). In addition, the FCI A group had significantly more medially located FHCs (*p* < 0.001). The differences between the two groups and the values for the entire study population are shown in Fig 1. For better visualization, the values of the parameters of the FCI A and FCI B hips are summarized in Table 2.

**Table 2:** The values (mean ± SD) of the parameters of the FCI A and B hips at early evaluation.

	FCI A	FCI B	*p*
**AR (**°)	4.42 ± 6.0	7.62 ± 7.2	<0.001
**AS (**°)	0.45 ± 1.9	1.55 ± 3.8	<0.001
**LI**	0.32 ± 0.1	0.36 ± 0.1	<0.001
**DARS (**°)	3.30 ± 1.8	3.77 ± 1.9	0.01
**FHC, n (%)**			
medial	278 (91.7)	130 (81.8)	<0.001
superimposed	24 (7.9)	28 (17.6)	
lateral	1 (0.3)	1 (0.6)	
**TS**	11.47 ± 8.3	16.65 ± 10.9	<0.001

No significant correlation was found between age and any of the parameters according to FCI grade.

### Differences between the breeds

The Labrador Retrievers and Golden Retrievers showed significant differences in all the parameters between the FCI grades. In particular, the FCI B group had significantly higher AS in both breeds (*p* = 0.03 and *p* = 0.04, respectively), LI (*p* = 0.01 and *p* = 0.01, respectively), and TS (*p* < 0.001 and *p* = 0.03, respectively). Among the Labrador Retrievers, the FCI B group had significantly higher AR and DARS (both *p* < 0.001). The data of the different breeds and their parameters are summarized in Table 3.

**Table 3:** Descriptive statistics of the evaluated parameters for the breeds with >10 animals.

	Border Collie		Bernese Mountain Dog		Golden Retriever		Labrador Retriever	
FCI grade (n)	A (42)	B (30)	A (49)	B (13)	A (32)	B (40)	A (137)	B (49)
**AR (°)**	4.74 ± 5.8	5.63 ± 5.6^**b**^	1.86 ± 3.9^**c**^	2.69 ± 4.8^**c**^	4.06 ± 5.9	6.48 ± 7.0^**d**^	6.05 ± 6.7^**c**^	11.80 ± 7.4^**a**^^**b**^^**c**^^**d**^
**AS (°)**	0.38 ± 1.6	0.57 ± 2.8	0.49 ±2.3	0.77 ± 2.8	0.53 ± 2.7	2.65 ± 5.5^**a**^	0.57 ± 1.9	1.67 ± 3.4^**a**^
**LI**	0.30 ± 0.1	0.33 ±0.1	0.31 ± 0.1	0.34 ± 0.1	0.33 ± 0.1	0.38 ± 0.1^**a**^	0.33 ± 0.1	0.37 ± 0.1^**a**^
**DARS (°)**	2.67 ± 1.7^**b**^	2.87 ± 1.1^**b**^	2.82 ± 1.7^**c**^	3.15 ± 1.4	3.31 ± 1.7	3.85 ± 2.2	3.87 ± 1.7^**b**^^**c**^	4.76 ± 2.1^**a**^^**b**^
**TS**	10.86 ± 7.0	12.50 ± 7.2^**b**^	8.39 ± 7.0^**c**^	10.08 ± 7.4^**c**^	11.28 ± 8.0	16.80 ± 12.1^**a**^	13.94 ± 9.4^**c**^	22.08 ± 11.3^**a**^^**b**^^**c**^
**CFH (n, %)**								
**medial**	40 (95.2)	28 (93.3)	44 (89.8)	12 (92.3)	30 (93.8)	31 (77.5)	121 (88.3)	35 (71.4)
**acetabular rim**	2 (4.8)	2 (6.7)	5 (10.2)	1 (7.7)	2 (6.3)	9 (22.5)	15 (10.9)	13 (26.5)
**lateral**	-	-	-	-		-	1 (0.7)	1 (2.0)

^**a**^ **Significant within-breed differences in the parameters.**

^**b**^ **Significant differences between the Border Collies and the Labrador Retriever**

^**c**^ **Significant differences between the Bernese Mountain Dog and the Labrador Retriever**

^**d**^ **Significant differences between the Golden Retriever and the Labrador Retriever.**

The Border Collie group showed negative correlations between age and AR (r = −0.33, *p* = 0.03) and TS (r = −0.34, *p* = 0.027) in the FCI A group. By contrast, the Labrador Retrievers showed positive correlations between age and AR (r = 0.20, *p* = 0.02) and TS (r = 0.17, *p* = 0.04) in the FCI A group. Only the Golden Retrievers showed a positive correlation between age and LI (r = 0.44, *p* < 0.001) in the FCI B group. No other relevant correlations were found.

The between-breed comparisons in the FCI A group showed that the AR and TS of the Labrador Retrievers were significantly higher than those of the Bernese Mountain Dogs (*p* < 0.001). DARS was also significantly higher in the Labrador Retrievers than in the Border Collies and Bernese Mountain Dogs (*p* < 0.001). The between-breed comparison in the FCI B group showed that AR was significantly higher in the Labrador Retrievers than in all the other three breeds (*p* < 0.001), and that DARS and TS were significantly higher in the Labrador Retrievers than in the Border Collies and Bernese Mountain Dogs (*p* < 0.001).

### Probability of prediction of FCI A and B hips

**Fig 2:**
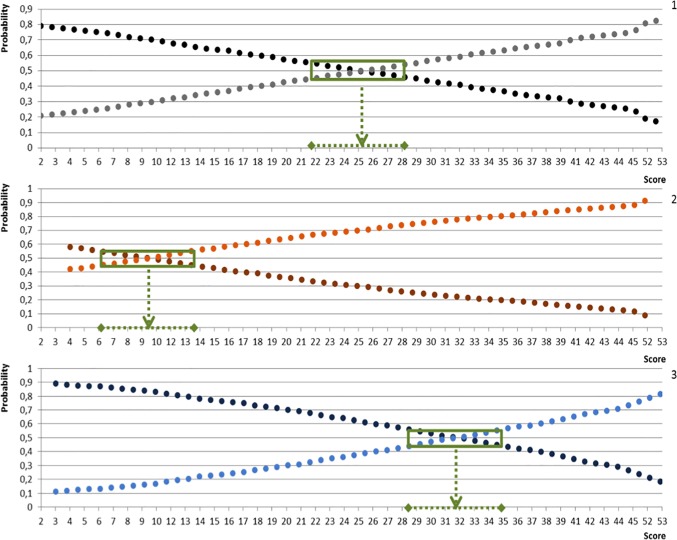
Probability curves of the entire population, Golden Retriever, and Labrador Retriever. Probability graph for the entire population (1), Golden Retrievers (2), and Labrador Retrievers (3): The dark and light dotted lines represent the probabilities for the development of FCI A and B hips, respectively. The green square marks the zone in which the probability is between 45% and 55%. In this zone, the score range from 22 to 28 for the entire population, from 6 to 14 for the Golden Retrievers, and from 28 to 35 for the Labrador Retrievers.

Fig 2 shows the predicted probabilities for FCI A or B hips according to the TS of the whole cohort and separately for the two breeds that showed significant differences in TS, namely the Labrador and Golden Retriever. For the entire cohort, TS had a high influence on the final FCI grade (*p* < 0.001), with an odds ratio (OR) of 1,066. In other words, the increment in TS by 1 increased the possibility of an FCI B hip by a factor of 1,066. The OR was significant for the Labrador Retriever at 1,085, but not for the Golden Retriever at 1,063, which can be an effect of the small sample size. Table 4 provides the results of the logistic regression analysis in more detail.

**Table 4:** Results of the logistic regression analysis for the entire cohort, Golden Retriever, and Labrador Retriever.

				95% Confidence Interval		
	intercept	slope	OR	lower	upper	p
**Cohort**	−1.36	0.064	1.066	1.037	1.097	<0.001
**Golden Retriever**	−0.48	0.061	1.063	0.991	1.139	0.081
**Labrador Retriever**	−2.273	0.082	1.085	1.036	1.136	<0.001

Fig 2 highlights the TS range for which prediction is difficult and wherein A and B overlap. This uncertain range is differs when considering the entire cohort (TS, 22–28), Labrador Retrievers (TS, 28–35), and Golden Retrievers (TS, 6–14).

## Discussion

The objective of this study was to verify whether EE can reveal differences in the evaluated parameters in skeletally immature hips [41] that are classified as FCI A and B in adult dogs. The entire multi-breed cohort and Labrador Retriever breed group showed that hip joints classified as FCI B had significantly higher AS, AR, LI, DARS, and TS at EE. The Golden Retriever breed group showed significantly higher AS, LI, and TS at EE only for the FCI B hips. As only normal and near-normal hips were evaluated, only minimal differences were expected. Another key finding of our study was that for the entire cohort, the FHC was significantly more often located medial to the acetabular rim in the FCI A hips.

The study also evaluated the influences of age and breed on the different parameters, and showed that in the entire cohort, age had no influence. In the breed groups, some influences were observed for both FCI grades. In the Border Collie FCI A subgroup, the correlations of age to AR and TS were negative, possibly because the breed is not highly predisposed to CHD. Meanwhile, with increased age, the AR and TS of the subgroup seemed to be decreased, showing a lower score in the measurements performed to quantify the joint laxity. To the best of our knowledge, no study has been conducted on CHD predisposition in this particular breed. By contrast, the Labrador Retriever FCI A subgroup showed positive correlations between age, and AR and TS, possibly reflecting the predisposition of this breed to an increased hip laxity and, subsequently, to CHD. The Golden Retriever FCI B subgroup showed a positive correlation between age and LI. This is contrary to the prevailing logic that age and LI should be negatively correlated, as hip laxity decreases over time due to capsular tightening and fibrosis [4,42], but is in concordance with a recent study [43]. The short period between the EE and the final examination in this study did not provide enough time for the development of capsular tightening of fibrosis [44]. An alternative explanation would be that in juvenile patients with a transitional coxofemoral hip joint, synovitis (even at low severity) can lead to increased laxity [3,4]. These aspects should be considered in subsequent studies with high number of cases and precise analysis of the broad array of etiological factors (i.e., muscle mass, growth rate, and environmental factors). Similar to our findings that age has a different correlation to laxity, a recent study [43] showed significant variations in DI values in dogs between 4, 6, and 12 months of age. Taroni et al. found that this parameter increased between 4 and 6 months of age but decreased between 6 and 12 months of age.

The most noteworthy differences observed in the evaluated parameters in this study were between the Labrador Retriever and Golden Retriever breed groups. This could be an artifact of our study cohort’s overrepresentation of the former breed; however, the latter breed is similar in size to the other two breeds examined (Bernese Mountain Dog and Border Collie). Nonetheless, the differences observed between the measured parameters in all the breeds were statistically relevant for hips that were not significantly different from a pathological standpoint (i.e., normal and near-normal hips) and indicate the importance of differentiating between breeds. It is still intriguing that Labrador Retrievers showed distinctive differences, which may indicate that EE prediction is more readily adoptable to this breed in particular.

The features of FCI A and B hips are very similar, which makes the distinction between them difficult. Our results suggest that using a TS instead of the single parameters in the EE, seems to improve the possibility of differentiating between these two grades according to differences in the probability curves constructed for the entire cohort, Golden Retriever, and Labrador Retriever. This is in accordance with a previous study [19] that applied the parameters to a global predictive score. Collectively, these findings confirm that several parameters are needed to predict CHD development [15,18,19,31,38,43,45]. Similarly to the previous study [19], our stud demonstrated that the TS summarized all the measured values and, up to a certain score, showed a predictive ability. The TS range where the probability was almost equal for predicting FCI A and B hips represents its weakness. This range was different for the entire cohort, Labrador Retrievers, and Golden Retriever. For the entire cohort and Labrador Retriever group, the distinctive range is sufficiently large to predict an FCI A hip; for Golden Retrievers, the indistinctive range is large, leaving only a small range for accurate prediction of the development of FCI A hip. Thus, for the Golden Retrievers, either differentiating between FCI A and FCI B is not possible when the TS is low or only prediction of FCI B or worse is possible when the TS is >20; prediction of an FCI A hip is uncertain with a low TS. This result also agrees with the previous study wherein the probability curves were calculated for all FCI grades [19]. A recent study showed that measurements in the distraction view, performed in the EE, can predict, FCI A, B or C in adult dogs [31], but are not accurate predictors of FCI grade at 12 months of age. In this study, 96% of the hips with a distraction index of <0.58 at 4 months of age were given FCI grades of A, B, or C FCI at 12 months of age.

Our results that show higher parameter values for FCI B hips correspond to previously published results [18,19]. However, some of the values we obtained at EE for FCI A and FCI B hips, such as the mean AR of 4.42° ± 6.0° and 7.62° ± 7.2°, respectively, were generally lower than the AR of 15° in the previous study [19]. The previous study used a remarkably smaller group size [4–11 dogs] and excluded dogs that tested negative for the Ortolani sign from the statistical analysis. The mean AS in our study were analogous to those described in the previous study [19], and the mean LI in our study were similar to the DI values published in other studies [19,46]. For LI, our findings not only confirmed the conclusion of Smith et al. that dogs with a DI of <0.3 rarely develop degenerative joint diseases and those with a DI of >0.3 may or may not develop degenerative joint diseases [38] but also agree with a recent study that established that a threshold of 0.58 for DI can accurately predict FCI A, B, and C hips [31]. The LI values in the present study can be compared with the DI values in the previous studies, as a similar interobserver agreement could have been demonstrated [33]. Another study [47] which included 313 dogs, found a low-to-moderate correlation between the results of Ortolani sign and the FCI hip grade. The Ortolani sign was tested positive in 31 (12.3%) of 252 dogs that were classified as having a FCI A or B hip. In contrast to our study, the previous study only used the Ortolani sign as a qualitative measurement, without quantifying the resulting laxity in the AR. The dogs tested for the Ortolani sign were ≥12 months of age in the previous study, whereas the dogs subjected to measurements in our study had not reached skeletal maturity.

Our study has limitations that may have affected our findings. These principally include the non-uniform distributions of the FCI A and B hips among the groups, with a greater number of FCI A hips, and the breeds assessed (particularly the considerably high number of Labrador Retrievers) and their FCI A and B hips within each group. The clear results for the prediction of final FCI A and B grades and overall differences in parameters between FCI A and FCI B in the Labrador Retriever group indicate the need for studies with large homogeneous groups to determine the standard parameter values. The long time span from the EE in this study, between 3 and 7 months of age, could be a limitation, although it reflects the normal clinical routine. However, age did not influence the parameters in our study, with the exception of the Golden Retrievers with FCI B hips. For a precise estimation of the influence of age, further study is needed in a wider range of dog breeds at different life stages and groups sufficiently sized for statistical analysis. Some other limitations were due to the retrospective nature of the study, such as the fact that the measurements were not performed by a single investigator. Nevertheless, LI measurement has shown good repeatability and reproducibility, with low intraobserver and interobserver variability [48,49].

Our results indicate that even in normal and near-normal hips, breeds showed significant differences, which could reflect the predisposition of some breeds for a higher hip laxity during early age that can lead to degenerative joint diseases [2,50]. Although previous studies compared breeds with a higher risk of CHD with those that are known to have low incidence rates of CHD [39,51], more studies are needed to establish clear values for the different breeds.

## Conclusions

If the values of the parameters used in the EE are converted into a TS, they can provide a predictive value, but care must be taken, as the TS is highly dependent on the breed. Therefore, no recommendations for breeds in general can be made. Further studies covering more breeds and larger sample sizes are needed to establish a general statement about the relevance of each parameter regarding individual breeds. Based on the results of our study, we can only recommend the use of several parameters to examine hip laxity in growing dogs.
